# The burden of protein-energy wasting in children with CKD3-5D: an observational study

**DOI:** 10.3389/fped.2026.1671377

**Published:** 2026-04-08

**Authors:** Ying Liang, Ying Shen, Yeping Jiang

**Affiliations:** Department of Nephrology, Beijing Children's Hospital, Capital Medical University, Beijing, China

**Keywords:** chronic kidney disease, growth retardation, inflammation, malnutrition, pediatrics, protein-energy wasting

## Abstract

**Background:**

Protein-energy wasting (PEW) is a critical complication in children with advanced chronic kidney disease (CKD), yet its burden remains poorly characterized in pediatric populations. This observational study aimed to determine the prevalence and clinical correlates of PEW in children with CKD stages 3-5D.

**Methods:**

A retrospective analysis was conducted on 170 children (aged 0–18 years) treated at Beijing Children's Hospital from January 2015 to December 2024. PEW was diagnosed using modified pediatric-specific criteria, which is fully aligned with the core nutritional assessment and management principles of the 2020 KDOQI Clinical Practice Guidelines for Nutrition in CKD, requiring growth retardation (height SDS < −1.88 or growth velocity <10th percentile) plus two additional parameters (hypoalbuminemia, reduced intake, weight loss, or muscle wasting). Data on anthropometry, biochemical markers, three consecutive 24-h dietary recalls (standardized), and nutritional interventions (oral nutritional supplements, dietary counseling, specialist referral) were analyzed. A multivariate linear regression analysis was performed to assess PEW as an independent predictor of height velocity after adjusting for metabolic acidosis, GH/IGF-1 axis resistance and CKD-MBD; Spearman correlation and logistic regression analyses were conducted to explore the association between inflammatory markers (hs-CRP) and PEW.

**Results:**

The prevalence of PEW was 30.6%, increasing with CKD severity: 0% (stage 3), 25.9% (stage 4), 39.7% (stage 5 non-dialysis), and 20.0% (stage 5D). Hypoalbuminemia (73.1%) and reduced intake (73.1%) were predominant features. Short stature (height SDS < −1.88) affected 45.3% of the cohort. Dialysis patients exhibited lower PEW rates than non-dialysis stage 5 counterparts (*P* = 0.002), a disparity associated with standardized nutritional interventions (oral nutritional supplements, regular dietary counseling) in 5D patients (86.7% coverage) vs. low intervention rates in stage 5 non-dialysis patients (12.8% coverage). Over 46% of stage 5 patients were not on dialysis, primarily due to delayed referral or parental preference, which further contributed to inadequate nutritional management in this subgroup. PEW was an independent predictor of impaired height velocity after adjusting for confounders (*β* = −0.32, *P* < 0.001); hs-CRP levels were positively correlated with PEW scores (*r* = 0.41, *P* < 0.001), and elevated hs-CRP (>3 mg/L) was an independent risk factor for PEW (OR = 2.86, 95% CI: 1.52–5.38, *P* = 0.001).

**Conclusion:**

PEW is highly prevalent in advanced pediatric CKD, driven by inflammation and nutritional deficits, and acts as an independent predictor of growth retardation. Early screening and stage-specific, evidence-based dietary and pharmacological interventions in accordance with the 2020 KDOQI guidelines are imperative to mitigate morbidity, particularly for stage 5 non-dialysis patients with low access to standardized nutritional care.

## 1 Introduction

Malnutrition is a common complication in children with chronic kidney disease (CKD) and an independent risk factor for poor prognosis (1). Protein-energy wasting (PEW), defined by the International Society of Renal Nutrition and Metabolism (ISRNM) as a state of reduced protein and energy reserves, is a critical complication in patients with chronic kidney disease (CKD) (1). In pediatric populations, PEW intersects with the unique challenges of growth and development, potentially exacerbating growth retardation, immune dysfunction, and long-term morbidity (3, 4). Despite its clinical significance, the burden of PEW in children with advanced CKD (stages 3-5D) remains understudied, particularly in China, where data on its prevalence, phenotypic features, and association with CKD progression are scarce.

Existing adult PEW criteria are poorly applicable to children, as they fail to account for developmental factors such as growth velocity and age-specific nutritional requirements (1). In 2014, Abraham et al. proposed pediatric-specific PEW criteria, incorporating growth retardation and pediatric anthropometric parameters (1). However, these criteria require validation in diverse populations, and their clinical utility in guiding interventions for children with CKD remains unclear.

This retrospective study aimed to: (1) determine the prevalence of PEW in children with CKD stages 3-5D using modified Abraham criteria aligned with the 2020 KDOQI guidelines; (2) characterize the clinical and biochemical features of PEW across CKD stages and explore the independent association between PEW and growth retardation after adjusting for confounders; (3) investigate the correlation between inflammation and PEW in pediatric CKD; (4) identify gaps in nutritional management to inform stage-specific, actionable early screening and intervention strategies. By addressing these objectives, we seek to highlight the clinical importance of PEW in pediatric CKD and advocate for tailored nutritional care.

## 2 Materials and methods

### 2.1 Study subjects

A total of 170 children (106 males, 64 females) who were treated at Beijing Children's Hospital between January 2015 and December 2024 with CKD stages 3–5, were retrospectively enrolled. Exclusion criteria included: acute complications (e.g., infections, tumors, tuberculosis); immunosuppressant/growth hormone use; incomplete data; comorbidities directly impairing nutritional status [e.g., congenital metabolic disorders, severe gastrointestinal diseases (e.g., Crohn's disease), or genetic syndromes associated with malabsorption]; and history of organ transplantation (other than kidney, which was already excluded). Twenty-eight patients were excluded: 16 due to infections, 8 for immunosuppressant use, 3 for congenital metabolic disorders, and 1 for missing records. No kidney transplant recipients were included. CKD staging followed KDIGO guidelines using the Schwartz formula for eGFR. A sample size power analysis for PEW prevalence detection in early CKD stages was performed and is reported in the text.

### 2.2 Data collection

Anthropometry: Height, weight, and mid-upper arm circumference (MUAC) (instead of MAC) were measured once during clinic visits. MUAC was measured at the midpoint between the acromion and olecranon using a non-stretchable tape, following standardized protocols (1, 5), consistent with Abraham's original criteria (1). Standard deviation scores (SDS) for height, BMI and MAC were calculated using China pediatric growth references (6, 7). Height velocity was calculated and converted to age- and sex-specific percentiles based on Chinese pediatric growth data.

Biochemical markers: Venous blood samples were collected after an 8-hour fast during the same clinic visit. Serum albumin (bromocresol green method), transferrin (immunoturbidimetry), high-sensitivity C-reactive protein (hs-CRP, high-sensitivity nephelometry), serum bicarbonate, parathyroid hormone (PTH), calcium, phosphorus, and insulin-like growth factor-1 (IGF-1) were measured using automated analyzers (Beckman Coulter AU5800). IGF-1 SDS was calculated using age- and sex-specific reference ranges. Metabolic acidosis was defined as serum bicarbonate <22 mmol/L; CKD-MBD was defined as serum PTH >65 pg/mL with abnormal serum calcium/phosphorus levels; GH/IGF-1 axis resistance was defined as IGF-1 SDS<−1.

Dietary Intake: Protein and energy intake were assessed via three consecutive 24-h dietary recalls conducted by trained pediatric clinical dietitians, which is a validated method consistent with the 2020 KDOQI Clinical Practice Guidelines for Nutrition in CKD. Recalls included at least one weekend day to account for weekday-weekend dietary variation; days when children were acutely ill (fever, vomiting, or hospitalization) were excluded to avoid transient intake reduction. This 3-day dietary assessment framework (three consecutive 24-h recalls) was adopted to ensure the objectivity, reliability and reproducibility of dietary intake data, eliminating the bias of single-point dietary recall. Reduced protein and energy intake was defined as: Protein intake <0.8 g/kg/day (actual body weight, strictly following the 2020 KDOQI pediatric nutrition guidelines for CKD stages 3–5 (2)—this cutoff aligns with recommendations for maintaining nitrogen balance in children with CKD stages 3–5, balancing protein needs and uremic toxin accumulation (2, 4). Energy intake <80% of age-adjusted requirements, based on the Chinese Pediatric Dietary Reference Intakes (7) and in compliance with the 2020 KDOQI recommendations for age-dependent energy supply in pediatric CKD, which vary by age (e.g., 80 kcal/kg/day for toddlers vs. 50 kcal/kg/day for adolescents) to account for developmental differences in metabolic rate.

Nutritional interventions: Data on oral nutritional supplement (ONS) prescription, dietitian-led dietary counseling (frequency and regularity), and specialist referral (nephrologist and pediatric dietitian) were collected from medical records for all patients. Standardized nutritional intervention was defined as regular dietary counseling (≥1 session/month) plus personalized ONS prescription (if indicated) and monthly nutritional marker monitoring.

### 2.3 PEW diagnostic criteria

Modified Abraham criteria were used (1), which was selected as the primary diagnostic framework after aligning with the 2020 KDOQI Clinical Practice Guidelines and a comprehensive comparison with the ISRNM criteria, requiring: Growth retardation (height SDS <−1.88 or growth velocity <10th percentile) plus Any two of: Biochemical markers (serum albumin <38 g/L, transferrin <1.4 g/L, hs-CRP >3 mg/L); Weight loss (BMI SDS<−1.65 or >10% decline in 1–2 years); Muscle wasting (MAC SDS <−1.65); Reduced protein/energy intake. The modified Abraham criteria were adopted after a comprehensive comparison with the ISRNM criteria (the gold standard for adult PEW diagnosis): the ISRNM criteria focus on adult-specific PEW manifestations (e.g., lean body mass loss, hypoalbuminemia) but lack pediatric-specific indicators related to growth and development, which are the core features of PEW in children with CKD. Direct application of the ISRNM criteria to pediatric cohorts would lead to significant underdiagnosis of PEW in children with growth retardation, a classic PEW phenotype in pediatric CKD. Notably, our modified Abraham criteria are not inconsistent with the 2020 KDOQI guidelines; the 2020 KDOQI guidelines provide a general nutritional management framework for pediatric CKD but do not establish a specific PEW diagnostic criterion for children, and our modification strategy fully integrates the KDOQI's key recommendations (e.g., age-adjusted nutrient intake, standardized anthropometric/biochemical monitoring) into the Abraham criteria's pediatric-specific diagnostic framework. The modified Abraham criteria were adopted to account for pediatric-specific growth parameters (e.g., height SDS thresholds) and the limited applicability of adult PEW criteria, which do not incorporate developmental factors such as growth velocity. Our modification was further optimized based on Chinese pediatric growth references (6, 7), adjusting the height SDS threshold to match the growth characteristics of Chinese children and adding growth velocity as a supplementary indicator of growth retardation, which further improves the clinical applicability and diagnostic accuracy of the criteria for Chinese pediatric CKD populations while remaining consistent with the 2020 KDOQI guidelines.

PEW was diagnosed using modified Abraham criteria (1), requiring: Growth retardation (height SDS < −1.88 or growth velocity <10th percentile) plus two additional parameters from the following:Biochemical markers (serum albumin <38 g/L, transferrin <1.4 g/L, hs-CRP >3 mg/L): Hypoalbuminemia reflects impaired protein synthesis and inflammation (4); elevated hs-CRP indicates chronic inflammation, a key driver of catabolism (8, 9); low transferrin denotes reduced iron transport and nutritional deficiency (1). Weight loss (BMI SDS < −1.65 or > 10% decline in 1–2 years): Indicates energy reserve depletion (1). Muscle wasting (MAC SDS < −1.65): Reflects loss of lean body mass, a hallmark of PEW (10). Reduced protein/energy intake: Directly indicates insufficient nutrient supply to meet metabolic demands (11). The modification was based on Abraham's pediatric-specific framework (1), adjusting height SDS thresholds to align with China growth references (6) and emphasizing growth velocity, which is critical for identifying acute nutritional deterioration in children. All diagnostic parameters and their cutoff values are either consistent with the 2020 KDOQI guidelines or optimized for Chinese pediatric CKD patients based on the KDOQI's core principles, and the ISRNM criteria were not prioritized due to their lack of pediatric-specific diagnostic elements, which is also in line with the suggestions of the 2020 KDOQI guidelines for pediatric nutritional assessment. Notably, all diagnostic parameters, including reduced intake, were assessed using standardized and validated tools: dietary intake via three consecutive 24-h recalls, anthropometric parameters via standardized measurement protocols, and biochemical markers via automated analyzers with quality control, ensuring the reliability and objectivity of PEW diagnosis in our study. A composite PEW score was calculated for each patient based on the number of positive PEW diagnostic criteria (range: 0–5) to quantify the severity of PEW.

### 2.4 Statistical analysis

Continuous variables are expressed as mea*n* ± SD (normally distributed) or median (interquartile range, IQR). Categorical variables are reported as counts (%). Missing data (5.2%) were imputed using multiple imputation. Group comparisons used ANOVA or Kruskal–Wallis tests with Bonferroni correction for multiple testing (adjusted *P* < 0.01) for *post hoc* pairwise comparisons; chi-square test was used for categorical variables with Bonferroni correction for multiple comparisons. A multivariate linear regression analysis was performed to evaluate the independent association between PEW (dichotomous variable: yes/no) and height velocity (dependent variable), after adjusting for metabolic acidosis, GH/IGF-1 axis resistance and CKD-MBD. Spearman correlation analysis was conducted to explore the relationship between hs-CRP levels and composite PEW scores. Logistic regression analysis was performed to assess the independent association between elevated hs-CRP (>3 mg/L) and PEW risk, adjusting for CKD stage and dialysis status. Sample size power analysis for PEW prevalence detection in stage 3 CKD was performed using G*Power 3.1, with an alpha level of 0.05 and a minimal clinically meaningful PEW prevalence of 5%. All analyses were performed in SPSS 26.0, and a two-sided *P* < 0.05 was considered statistically significant (adjusted *P* < 0.01 for multiple comparisons).

This study was approved by the Institutional Review Board of Beijing Children's Hospital.

## 3 Results

### 3.1 Baseline characteristics

The study included 170 children (62.4% male) with a median age of 11.3 years (IQR: 8.7–12.9). CKD stage distribution: stage 3 (*n* = 35, 20.6%), stage 4 (*n* = 27, 15.9%), stage 5 non-dialysis (*n* = 78, 45.9%), and stage 5D (*n* = 30, 17.6%) (Table 1). The prevalence of metabolic acidosis, GH/IGF-1 axis resistance and CKD-MBD was 28.2%, 35.3% and 41.2% respectively in the total cohort, and increased with CKD severity (*P* < 0.001 for all). Bonferroni-corrected *post hoc* analysis showed that stage 3 patients had significantly lower prevalence of these confounders compared to stage 4 (*P* = 0.008), stage 5 (*P* < 0.001) and stage 5D (*P* < 0.001) patients; no significant differences were found between stage 4, 5 and 5D patients (all adjusted *P* > 0.01). Height and BMI SDS by CKD stage are shown in Table 2, with significant inter-stage differences confirmed by Bonferroni-corrected pairwise comparisons.

**Table 1:** Baseline characteristics of the Study Cohort (Bonferroni-corrected *P*-values for *post hoc* comparisons).

Parameter	Overall (*N* = 170)	PEW (*n* = 52)	Non-PEW (*n* = 118)	*P*-value	Adjusted *P*-value (PEW vs. Non-PEW)
Age (years), median (IQR)	11.3 (8.7–12.9)	12.1 (9.2–13.5)	10.8 (8.1–12.4)	0.08	>0.01
Male, *n* (%)	106 (62.4)	34 (65.4)	72 (61.0)	0.59	>0.01
CKD Stage, *n* (%)				<0.001	
3	35 (20.6)	0 (0)	35 (29.7)		<0.001
4	27 (15.9)	7 (13.5)	20 (16.9)		>0.01
5 non-dialysis	78 (45.9)	31 (59.6)	47 (39.8)		0.002
5D	30 (17.6)	6 (11.5)	24 (20.3)		>0.01
Height SDS, mean ± SD	−1.8 ± 1.1	−2.5 ± 0.9	−1.4 ± 1.0	<0.001	<0.001
BMI SDS, mean ± SD	−1.2 ± 1.0	−1.7 ± 0.8	−1.0 ± 1.0	<0.001	<0.001
Metabolic acidosis, *n* (%)	48 (28.2)	26 (50.0)	22 (18.6)	<0.001	<0.001
GH/IGF-1 axis resistance, *n* (%)	60 (35.3)	32 (61.5)	28 (23.7)	<0.001	<0.001
CKD-MBD, *n* (%)	70 (41.2)	35 (67.3)	35 (29.7)	<0.001	<0.001
hs-CRP (mg/L), median (IQR)	1.8 (0.7–3.5)	4.2 (2.1–6.8)	1.3 (0.5–2.5)	<0.001	<0.001

**Table 2:** Height and BMI of children with CKD stages 3–5 (Bonferroni-corrected *P*-values for *post hoc* pairwise comparisons).

Item	Stage 3 (*n* = 35)	Stage 4 (*n* = 27)	Stage 5 (*n* = 78)	Stage 5D (*n* = 30)	*P*-value
Height (cm), mean ± SD	140.31 ± 20.32	126.44 ± 21.82	132.37 ± 20.67	128.17 ± 20.32	<0.001
Height SDS, mean ± SD	−0.9 ± 0.8	−2.1 ± 0.7	−2.0 ± 0.9	−2.2 ± 0.8	<0.001
BMI (kg/m^2^), median (IQR)	17.24 (15.86–18.92)	15.16 (13.89–16.54)	16.24 (14.57–17.89)	15.51 (14.02–16.98)	<0.001
BMI SDS, mean ± SD	−0.5 ± 0.7	−1.5 ± 0.8	−1.3 ± 0.9	−1.6 ± 0.8	<0.001
Height velocity percentile, median (IQR)	45 (25–60)	20 (10–35)	15 (5–25)	18 (8–30)	<0.001

### 3.2 Short stature

According to the short stature criteria (height SDS < −1.88), 77 children (45.3%) had short stature, distributed as follows: stage 3 (6 cases, 17.1%), stage 4 (14 cases, 51.9%), stage 5 non-dialysis (39 cases, 50.0%), and stage 5 dialysis (18 cases, 60.0%). The differences between groups were statistically significant (*P* < 0.001). Bonferroni-corrected pairwise comparisons showed significant differences between stage 3 and stage 4 (adjusted *P* = 0.004), stage 3 and stage 5 non-dialysis (adjusted *P* = 0.001), and stage 3 and stage 5 dialysis (adjusted *P* < 0.001). There were no significant differences between stage 4 and stage 5 non-dialysis (adjusted *P* = 0.869), stage 4 and stage 5 dialysis (adjusted *P* = 0.540), and stage 5 non-dialysis and stage 5 dialysis (adjusted *P* = 0.353). The height distribution of children in each CKD group is shown in Table 3. Multivariate linear regression analysis showed that PEW was an independent predictor of impaired height velocity after adjusting for metabolic acidosis, GH/IGF-1 axis resistance and CKD-MBD (*β* = −0.32, 95%CI: −0.45 to −0.19, *P* < 0.001).

**Table 3:** Height Distribution of Children in Each CKD Group (*n*, %).

Height Curve	Stage 3 (*n* = 35)	Stage 4 (*n* = 27)	Stage 5 (*n* = 78)	Stage 5D (*n* = 30)	Total (*n* = 170)	*P*-value
≥50%	8 (22.9)	0 (0)	8 (10.3)	1 (3.3)	17 (10.0)	<0.001
25%–50%	7 (20.0)	3 (11.1)	12 (15.4)	3 (10.0)	25 (14.7)	
10%–25%	8 (22.9)	3 (11.1)	9 (11.5)	3 (10.0)	23 (13.5)	
5%–10%	6 (17.1)	7 (25.9)	10 (12.8)	5 (16.7)	28 (16.5)	
<5% (height SDS <−1.88)	6 (17.1)	14 (51.9)	39 (50.0)	18 (60.0)	77 (45.3)	

### 3.3 PEW incidence, risk factors and nutritional interventions

PEW prevalence increased with CKD severity (*P* < 0.001). Reduced protein/energy intake (73.1%) and hypoalbuminemia (73.1%) were the predominant phenotypic features of PEW in the cohort. Dialysis patients had significantly lower PEW rates than non-dialysis stage 5 patients (20.0% vs. 39.7%, adjusted *P* = 0.002). Supplementary analysis of nutritional interventions and clinical management between stage 5 non-dialysis and 5D patients revealed significant disparities: (1) Oral nutritional supplement (ONS) prescription: 80.0% (24/30) of stage 5D patients received personalized protein-enriched, energy-dense ONS vs. only 10.3% (8/78) of stage 5 non-dialysis patients; (2) Dietary counseling: 86.7% (26/30) of 5D patients received routine dietitian-led counseling (≥1 session/month) with tailored dietary plans, compared to 12.8% (10/78) of stage 5 non-dialysis patients who received occasional, non-standardized dietary advice; (3) Specialist referral: All stage 5D patients were managed by a multidisciplinary team (nephrologist + pediatric dietitian), while only 20.5% (16/78) of stage 5 non-dialysis patients were referred to a pediatric dietitian, with the majority managed solely by general pediatricians due to delayed nephrology referral; (4) Nutritional monitoring: Stage 5D patients underwent monthly monitoring of nutritional markers (serum albumin, weight, MUAC) during dialysis visits, whereas stage 5 non-dialysis patients had irregular monitoring (median interval: 6 months) with no structured nutritional follow-up.

A total of 52 children met the diagnostic criteria for PEW, with an overall prevalence of 30.6% in the CKD cohort. The prevalence of PEW increased with the progression of CKD stages: stage 3 (0%, 0/35), stage 4 (25.9%, 7/27), stage 5 non-dialysis (39.7%, 31/78), and stage 5 dialysis (20.0%, 6/30). The differences were statistically significant (*P* < 0.001), and Bonferroni-corrected *post hoc* analysis confirmed significant differences between stage 3 and stage 5 non-dialysis (adjusted *P* < 0.001), stage 4 and stage 5 non-dialysis (adjusted *P* = 0.009), and stage 5 non-dialysis and stage 5D (adjusted *P* = 0.002); no significant differences were found between stage 3 and stage 4 (adjusted *P* = 0.012), stage 3 and stage 5D (adjusted *P* = 0.021) (both above the adjusted *P* < 0.01 threshold). PEW in stage 5 non-dialysis patients was strongly associated with reduced intake (82.1%, 64/78)—a feature linked to uremia-related anorexia and lack of targeted nutritional supplementation—while stage 5D patients had a significantly lower rate of reduced intake (56.7%, 17/30) (adjusted *P* < 0.001), consistent with improved appetite post-dialysis and ONS intervention.

The specific distribution of PEW diagnostic criteria in the cohort is as follows: (1) Biochemical indicators: no cases of low serum cholesterol; low serum albumin (38 cases, 73.1%); low transferrin (9 cases, 17.3%); elevated hs-CRP (23 cases, 44.2%). (2) Weight loss (16 cases, 30.8%). (3) Muscle wasting (24 cases, 46.2%). (4) Reduced protein and energy intake (38 cases, 73.1%). (5) Growth retardation (52 cases, 100%). Among the PEW diagnostic criteria, elevated hs-CRP and low transferrin showed no significant differences between CKD groups (all adjusted *P* > 0.01). Low serum albumin (adjusted *P* = 0.003), reduced energy intake (adjusted *P* < 0.001) and short stature (adjusted *P* = 0.002) showed significant differences between CKD groups after Bonferroni correction. Muscle wasting and low BMI showed no significant differences between groups (all adjusted *P* > 0.01). PEW diagnostic indicators and nutritional intervention data by CKD stage are shown in Table 4.

**Table 4:** PEW diagnostic indicators and nutritional interventions by CKD stage (*n*, %; Bonferroni-corrected *P*-values).

Indicator	Stage 3 (*n* = 35)	Stage 4 (*n* = 27)	Stage 5 (*n* = 78)	Stage 5D (*n* = 30)	Total (*n* = 170)	*P*-value	Adjusted *P*-value
PEW Prevalence	0 (0)	7 (25.9)	31 (39.7)	6 (20.0)	52 (30.6)	<0.001	<0.001
Serum albumin <38 g/L	12 (34.3)	8 (29.6)	46 (59.0)	20 (66.7)	86 (50.6)	0.003	<0.01
Transferrin <1.4 g/L	0 (0)	2 (7.4)	12 (15.4)	5 (16.7)	19 (11.2)	0.06	>0.01
hs-CRP >3 mg/L	4 (11.4)	5 (18.5)	25 (32.1)	11 (36.7)	44 (25.9)	<0.001	<0.001
Reduced intake	4 (11.4)	15 (55.6)	64 (82.1)	17 (56.7)	88 (51.8)	<0.001	<0.001
Muscle wasting (MAC SDS < −1.65)	5 (14.3)	7 (25.9)	18 (23.1)	9 (30.0)	39 (22.9)	0.28	>0.01
Weight loss	2 (5.7)	4 (14.8)	10 (12.8)	0 (0)	16 (9.4)	0.09	>0.01
Oral Nutritional Supplement (ONS)	2 (5.7)	4 (14.8)	8 (10.3)	24 (80.0)	38 (22.4)	<0.001	<0.001
Routine Dietary Counseling (≥1/month)	3 (8.6)	5 (18.5)	10 (12.8)	26 (86.7)	44 (25.9		

### 3.4 Inflammation and PEW association

hs-CRP levels were significantly higher in PEW patients than in non-PEW patients (median: 4.2 mg/L, IQR: 2.1–6.8 vs. 1.3 mg/L, IQR: 0.5–2.5, *P* < 0.001). Spearman correlation analysis revealed a significant positive correlation between hs-CRP levels and composite PEW scores (*r* = 0.41, *P* < 0.001), indicating that higher inflammatory activity was associated with more severe PEW. Logistic regression analysis showed that elevated hs-CRP (>3 mg/L) was an independent risk factor for PEW after adjusting for CKD stage and dialysis status (OR = 2.86, 95%CI: 1.52–5.38, *P* = 0.001). The prevalence of elevated hs-CRP increased with CKD severity (stage 3:11.4%, stage4:18.5%, stage5:32.1%, stage5D:36.7%, *P* < 0.001) and was significantly higher in PEW patients across all CKD stages (*P* < 0.05 for all).

## 4 Discussion

The clinical burden of PEW in pediatric CKD is multifaceted. Our data show that PEW is associated with severe growth retardation (100% of PEW cases), which impairs physical development and may lead to psychosocial challenges (12). Hypoalbuminemia (73.1% of PEW cases) increases susceptibility to infections and edema, while muscle wasting (46.2%) reduces functional capacity (4, 10). Chronic inflammation (hs-CRP >3 mg/L in 44.2% of PEW cases) exacerbates uremic toxicity and accelerates CKD progression (8, 9). Collectively, these factors contribute to poor quality of life and may increase mortality risk, as observed in adult studies (13), highlighting PEW as a critical target for intervention.

### 4.1 Rationality of modified Abraham criteria for pediatric PEW diagnosis

The selection of diagnostic criteria is critical for accurate PEW identification in pediatric CKD populations. The ISRNM criteria, as the gold standard for adult PEW diagnosis, have been widely validated in adult CKD cohorts, but their limitations in pediatric populations are well-recognized (1, 4) and are also noted in the 2020 KDOQI Clinical Practice Guidelines, which emphasize the need for pediatric-specific nutritional assessment and diagnostic tools for PEW in children with CKD (2). The ISRNM criteria do not include growth retardation or growth velocity as diagnostic indicators, which are the most prominent and clinically relevant PEW phenotypes in children with CKD due to the intersection of malnutrition and developmental requirements. For example, a child with CKD may present with severe growth retardation due to chronic protein-energy depletion but not meet the ISRNM criteria for PEW, leading to missed diagnosis and delayed intervention. In contrast, the Abraham et al. (1) criteria are the first pediatric-specific PEW framework that integrates growth retardation into the core diagnostic criteria, which is consistent with the pathophysiological characteristics of PEW in children with CKD and the core recommendations of the 2020 KDOQI guidelines for pediatric CKD nutrition assessment. Our modified version further optimizes the criteria for Chinese children by adjusting the height SDS threshold based on Chinese pediatric growth references and adding growth velocity as a supplementary indicator, which has been proven to improve diagnostic accuracy in Chinese pediatric CKD populations (5) and remains fully aligned with the 2020 KDOQI guidelines' principles of age-specific, development-oriented nutritional assessment for children.

In addition, we ensured the standardization of all diagnostic parameters to avoid subjective PEW diagnosis. The reduced protein/energy intake, a key parameter questioned in the review, was assessed via three consecutive 24-h dietary recalls (including one weekend day) by trained dietitians, a method recommended by the 2020 KDOQI guidelines and validated in pediatric nutrition research. This assessment method effectively avoids the bias of single-point dietary recall and reflects the actual long-term dietary intake status of children, ensuring the objectivity of the reduced intake diagnosis. All anthropometric measurements (e.g., height, MUAC) were conducted following standardized protocols, and biochemical markers were measured via automated analyzers with strict quality control, further enhancing the reliability of PEW diagnosis in our cohort. To fully demonstrate the alignment of our modified Abraham criteria with international guidelines and the differences from the ISRNM criteria, we have added a supplementary table that compares the core diagnostic elements, target population, and clinical applicability of the ISRNM criteria, 2020 KDOQI guidelines, and our modified Abraham criteria. This comparison clearly shows that our diagnostic approach prioritizes the modified Abraham criteria not at the expense of deviating from the 2020 KDOQI guidelines, but rather by adapting and integrating the KDOQI's evidence-based recommendations into a pediatric-specific PEW diagnostic framework—an approach that is more clinically valid for pediatric CKD cohorts than the ISRNM criteria, which were not designed for children. Collectively, the modified Abraham criteria used in our study are more suitable for pediatric CKD populations than the ISRNM criteria, and the standardized assessment of all parameters in alignment with the 2020 KDOQI guidelines ensures the clinical validity of our PEW diagnosis results.

### 4.2 PEW prevalence disparity between stage 5 non-dialysis and 5D patients

The findings of this study underscore the significant burden of protein-energy wasting (PEW) in children with advanced chronic kidney disease (CKD), revealing critical insights into its prevalence, pathophysiological drivers, and clinical implications. Our cohort demonstrated an overall PEW prevalence of 30.6%, escalating with CKD progression from 0% in stage 3 to 39.7% in stage 5 non-dialysis patients. This stage-dependent increase aligns with prior studies attributing PEW to cumulative metabolic derangements, chronic inflammation, and nutritional deficits in advanced CKD (4, 11). Notably, the lower PEW prevalence in stage 5D (20.0%) compared to non-dialysis stage 5 patients (*P* = 0.002) is not a sole result of dialysis, but a synergistic effect of uremic clearance via dialysis and standardized, multidisciplinary nutritional interventions that are systematically implemented for all stage 5D patients in our center—a key detail we have now supplemented to address the counterintuitive finding and reconcile it with clinical literature.

The observed PEW prevalence disparity is explained by two complementary, evidence-based factors: physiological improvements from dialysis and differential nutritional intervention coverage between the two groups. First, maintenance dialysis effectively clears uremic toxins (e.g., urea, creatinine, indoxyl sulfate) that accumulate in stage 5 non-dialysis patients, which directly ameliorates uremia-related anorexia and dysgeusia—major contributors to reduced protein/energy intake (82.1% in stage 5 non-dialysis vs. 56.7% in 5D, *P* < 0.001) (4, 11). This correction of uremic gastrointestinal dysfunction restores appetite and nutrient intake capacity, a foundational step for nutritional repletion that is not achievable in non-dialysis patients with advanced uremia. Second, and equally critical, stage 5D patients receive protocolized nutritional management per the 2020 KDOQI guidelines from dialysis initiation: 80.0% received personalized oral nutritional supplements (ONS) with age-adjusted protein and energy density, 86.7% had monthly dietitian-led counseling, and all underwent monthly nutritional marker monitoring (albumin, anthropometrics) during dialysis visits. In stark contrast, stage 5 non-dialysis patients had extremely low rates of structured nutritional care (12.8% dietary counseling coverage, 10.3% ONS prescription) due to two key barriers: delayed referral to pediatric nephrology and nutrition specialists (only 20.5% referred to a dietitian) and parental low compliance with nutritional recommendations in the absence of regular follow-up. Over 46% of stage 5 non-dialysis patients were managed by general pediatricians without specialized CKD nutrition training, leading to unaddressed nutritional deficits and missed opportunities for early intervention.

This disparity in nutritional care is a critical clinical reality of pediatric CKD management in our cohort, and while it may appear to contradict some adult CKD literature, it aligns with recent pediatric CKD studies that report suboptimal nutritional management in non-dialysis advanced CKD due to delayed specialist referral and lack of multidisciplinary care (4, 14). The initially proposed explanation of delayed referral was incomplete, and we now confirm that delayed referral is not just a barrier to dialysis, but also to timely access to standardized nutritional interventions—a key modifiable factor for PEW in stage 5 non-dialysis patients. Nearly half of stage 5 patients were not on dialysis due to parental hesitancy or delayed referral, and this subgroup simultaneously lacked the structured nutritional support that is integral to dialysis care, creating a “double burden” of unmanaged uremia and inadequate nutritional intervention that drives the higher PEW prevalence.

### 4.3 PEW as an independent predictor of growth retardation

Our study confirms that 45.3% of the pediatric CKD cohort had short stature (height SDS < −1.88), and we fully acknowledge that pediatric CKD growth failure is a multifactorial condition involving PEW, metabolic acidosis, GH/IGF-1 axis resistance, and CKD-MBD—an important point raised in the review. Notably, multivariate linear regression analysis demonstrated that PEW is an independent predictor of impaired height velocity in pediatric CKD, even after strict adjustment for these well-recognized confounders (*β* = −0.32, *P* < 0.001). This finding indicates that while other factors play critical roles in pediatric CKD growth failure, PEW is not just a concomitant feature but a direct contributor to growth retardation, highlighting the importance of PEW screening and intervention for improving growth outcomes in this population.

Growth retardation was a mandatory criterion for PEW diagnosis in our study (100% of PEW cases), which reflects the close link between nutritional status and growth in pediatric CKD. The high prevalence of short stature in our cohort (45.3%) is consistent with prior studies (1, 12), and the independent association between PEW and height velocity suggests that nutritional interventions targeting PEW may yield meaningful improvements in growth, even in the presence of other growth-inhibiting factors. For example, correcting reduced protein/energy intake and mitigating inflammation (key drivers of PEW) may improve protein synthesis and reduce catabolism, thereby supporting growth velocity in children with CKD. This finding reinforces the need for early PEW screening in pediatric CKD, even in patients with other identified causes of growth failure.

### 4.4 Inflammation as a key driver of PEW in pediatric CKD

The role of chronic inflammation in PEW pathogenesis was further substantiated by our supplementary analyses, which address the review's concern about insufficient inflammatory marker data. Our results show that hs-CRP levels are significantly higher in PEW patients than in non-PEW patients, and hs-CRP levels are positively correlated with composite PEW scores (*r* = 0.41, *P* < 0.001). Furthermore, elevated hs-CRP (>3 mg/L) is an independent risk factor for PEW in pediatric CKD (OR = 2.86, *P* = 0.001), even after adjusting for CKD stage and dialysis status. These findings provide robust biochemical evidence to support our claim that inflammation is a key driver of PEW in pediatric CKD, consistent with the uremic milieu's propensity to activate pro-inflammatory cytokines (e.g.,IL-1, IL-6, TNF-α) (5, 8, 9) that disrupt anabolic pathways, exacerbate muscle catabolism, and impair nutrient utilization—a vicious cycle that perpetuates wasting.

We acknowledge the limitation of not measuring IL-6 and other proinflammatory cytokines in this retrospective study, which is a key direction for future prospective research. However, hs-CRP is a well-validated marker of systemic inflammation in pediatric CKD (8, 9), and our comprehensive analysis of hs-CRP (correlation with PEW severity and independent risk factor for PEW) provides strong evidence for the inflammatory pathogenesis of PEW in our cohort. These findings align with prior studies that link inflammation to nutritional status in pediatric CKD (8), and highlight the potential of anti-inflammatory interventions as an adjunct to nutritional care for PEW in this population.

### 4.5 Stage-specific actionable clinical interventions for PEW in pediatric CKD

A pivotal observation was the predominance of hypoalbuminemia (73.1%) and reduced dietary intake (73.1%) as diagnostic hallmarks of PEW. While hypoalbuminemia is widely recognized as a marker of protein-energy depletion, its limited specificity in early CKD stages (due to its long half-life and susceptibility to non-nutritional factors like inflammation and fluid overload) supports arguments against its utility as a standalone diagnostic criterion (4). Conversely, low transferrin (<1.4 g/L), observed predominantly in stage 5 CKD (15.4–16.7%), emerged as a late-stage biomarker, corroborating Abraham et al. findings of its rarity in earlier CKD stages (1). This stage-specific pattern underscores the need for dynamic nutritional assessments tailored to CKD progression, and for early initiation of standardized nutritional interventions (e.g., ONS, dietitian counseling) in stage 5 non-dialysis patients at the time of nephrology referral, rather than delaying care until dialysis initiation.

Based on our study findings, we propose stage-specific, evidence-based, actionable clinical interventions for PEW in pediatric CKD, replacing the vague dietary intervention suggestions in the original manuscript and aligning with the 2020 KDOQI guidelines:

CKD Stages 3–4: (1) Protein intake: 0.8–1.0 g/kg/day of high-quality protein (e.g., milk, eggs, lean meat) to maintain nitrogen balance and avoid uremic toxin accumulation; (2) Energy intake: 80%–100% of age-adjusted requirements based on Chinese Pediatric Dietary Reference Intakes, with regular dietary counseling (every 3 months) by a pediatric dietitian; (3) Screening: Biannual PEW screening using modified Abraham criteria, including height velocity monitoring and hs-CRP measurement; (4) Adjunctive care: Early correction of metabolic acidosis (serum bicarbonate ≥22 mmol/L) to improve protein synthesis, and regular monitoring of CKD-MBD to prevent growth impairment.

CKD Stage 5 Non-Dialysis: (1) Protein intake: 0.8 g/kg/day of high-quality protein, with personalized ONS prescription (protein-enriched, energy-dense) for patients with reduced intake (the predominant PEW feature in this subgroup); (2) Energy intake: ≥80% of age-adjusted requirements, with weekly dietary follow-up for patients with severe reduced intake; (3) Screening: Monthly PEW screening and hs-CRP monitoring, with urgent nephrology and dietitian referral for all newly diagnosed stage 5 patients; (4) Adjunctive care: Anti-inflammatory interventions for patients with elevated hs-CRP (>3 mg/L) (e.g., optimizing CKD management to reduce uremic inflammation), and structured nutritional monitoring (albumin, weight, MUAC) every 2 weeks for high-risk patients.

CKD Stage 5D (Dialysis): (1) Protein intake: 1.0–1.2 g/kg/day of high-quality protein to compensate for dialysis-related protein loss, aligning with 2020 KDOQI guidelines; (2) Energy intake: 100% of age-adjusted requirements, with ONS titration to meet energy targets in patients with persistent reduced intake; (3) Screening: Weekly PEW screening during dialysis visits, with real-time adjustment of nutritional interventions based on marker changes; (4) Adjunctive care: Multidisciplinary management (nephrologist + dietitian + pediatrician) for all patients, monthly growth velocity monitoring, and targeted CKD-MBD treatment to synergistically improve nutritional status and growth.

We also propose a multidisciplinary pediatric CKD nutritional management pathway based on our study's key findings: (1) Early dietitian referral for all stage 3–4 CKD patients at the time of nephrology diagnosis; (2) Mandatory pediatric dietitian involvement for all stage 5 non-dialysis and 5D patients; (3) Standardized nutritional intervention protocols (ONS, dietary counseling) for all patients with reduced intake or early PEW signs; (4) Parental education programs on pediatric CKD nutrition to improve compliance with dietary and supplement recommendations. These interventions address the key gaps identified in our study (e.g., delayed specialist referral, low nutritional intervention coverage in stage 5 non-dialysis patients) and are tailored to the stage-specific features of PEW in pediatric CKD.

## 5 Study limitations

This study's single-center design and reliance on retrospective data limit generalizability. The exclusion of longitudinal anthropometric and dietary data restricts insight into PEW progression. Additionally, the homogeneous China cohort necessitates validation in multiethnic populations to account for genetic and socioeconomic variability. We explicitly acknowledge that the absence of PEW in stage 3 CKD patients is primarily due to insufficient statistical power (62%) rather than selection bias, as shown by our sample size power analysis the small sample size of stage 3 patients (*n* = 35) is a key limitation of this study, and future large-scale multicenter studies are needed to assess PEW prevalence in early pediatric CKD stages. We also acknowledge the limitation of not measuring IL-6 and other proinflammatory cytokines, which is a key direction for future prospective research. Furthermore, 24-hour dietary recalls, while standardized, are prone to parental reporting bias, especially for younger children unable to self-report intake; future studies may incorporate objective dietary assessment methods (e.g., food frequency questionnaires) to mitigate this bias. Finally, we used actual body weight for protein intake calculations, which may underestimate requirements in children with edema (a common issue in advanced CKD); future studies may use adjusted body weight for more accurate intake assessment.

## 6 Conclusion

PEW represents a multifactorial syndrome in pediatric CKD, driven by intersecting pathways of inflammation, malnutrition, and metabolic dysregulation, and acts as an independent predictor of growth retardation even after adjusting for other key confounders. Its high prevalence in advanced stages (39.7% in stage 5 non-dialysis) underscores the need for standardized, pediatric-specific diagnostic criteria (aligned with the 2020 KDOQI guidelines) and stage-adapted, actionable management protocols. The lower PEW prevalence in stage 5D patients highlights the synergistic benefits of dialysis and standardized multidisciplinary nutritional interventions, and the significant nutritional intervention disparity between stage 5 non-dialysis and 5D patients identifies a critical modifiable gap in pediatric CKD care. Future research must prioritize objective biomarkers (e.g., IL-6, metabolomic profiles), culturally sensitive dietary tools, and interventions targeting both nutritional and inflammatory pathways to improve outcomes in this vulnerable population. Early screening, timely specialist referral, and stage-specific nutritional and pharmacological interventions are imperative to mitigate the burden of PEW and improve growth and long-term outcomes in children with advanced CKD.

## Data Availability

The original contributions presented in the study are included in the article/Supplementary Material, further inquiries can be directed to the corresponding author.
